# Radial Scars and Subsequent Breast Cancer Risk: A Meta-Analysis

**DOI:** 10.1371/journal.pone.0102503

**Published:** 2014-07-14

**Authors:** Mengmeng Lv, Xingya Zhu, Shanliang Zhong, Weixian Chen, Qing Hu, Tengfei Ma, Jun Zhang, Xiaohui Zhang, Jinhai Tang, Jianhua Zhao

**Affiliations:** 1 Department of General Surgery, Nanjing Medical University Affiliated Cancer Hospital, Cancer Institute of Jiangsu Province, Nanjing, China; 2 The First Clinical School of Nanjing Medical University, Nanjing, China; 3 Gulou Clinical Medical College, Nanjing Medical University, Nanjing, China; 4 Center of Clinical Laboratory, Nanjing Medical University Affiliated Cancer Hospital, Cancer Institute of Jiangsu Province, Nanjing, China; The University of Hong Kong, Queen Mary Hospital, Hong Kong

## Abstract

**Background:**

The relationship between radial scars and breast cancer is unclear, as the results of different studies are inconsistent. We aim to solve the controversy and assess the breast cancer risk of radial scars.

**Methods:**

Case-control or cohort studies about radial scars and breast cancer risk published in PubMed, Web of Science and the Cochrane Library from 2000 to 2013 were searched. Heterogeneity for the eligible data was assessed and a pooled odds ratio (OR) with 95% confidence interval (CI) was calculated.

**Results:**

Five observational studies involving 2521 cases and 20290 controls were included in our study. From pooled analysis, radial scars were found to have a 1.33 fold increased risk of breast cancer, but which was not significant (*P* = 0.138). Sample size contributed to heterogeneity. In subgroup analysis, the results pooled from studies with sample size >2000 show that presence of radial scars was associated with 1.6 times breast cancer risk compared to absence of radial scars. Radial scars increased the risk of breast cancer among women with proliferative disease without atypia, but no significant association between radial scars and carcinoma was noted among women with atypical hyperplasia.

**Conclusions:**

Radial scars tend to be associated with an increased breast cancer risk. Radial scars should be considered among women with proliferative disease without atypia, while atypical hyperplasia is still the primary concern among women with both radial scars and atypical hyperplasia.

## Introduction

Benign breast diseases are classified into three categories by Dupont and Page: nonproliferative disease, proliferative disease without atypia (PDWA), and atypical hyperplasia (AH) [1]. This classification is commonly applied to examine the association between benign breast disease and breast cancer. Women with PDWA and with AH are at 1.5- to 2-fold, and 4- to 5-fold increased risk to develop breast cancer, respectively, compared to nonproliferative lesions [2]–[5]. However, the breast cancer risk of specific histologic features such as radial scar has not been well established [6].

Before the term “radial scar” appeared in 1980, this lesion was named in many other ways: sclerosing papillary proliferation, nonencapsulated sclerosing lesion, benign sclerosing ductal proliferation, or infiltrating epitheliosis [7], [8]. Microscopically, radial scars are characterized as a central fibroelastic core with radially arranged ducts and lobules [9]. Radial scars as benign proliferative lesion are similar to breast cancer in mammography, and the presence of carcinoma within some radial scars has also been reported [8], . The nature of radial scars remains unclear. Radial scars were suggested to be related to the histogenesis of breast cancer and may be a precursor [13], [14], but the opinions about this hypothesis are conflicting. In several autopsy studies, no difference in the presence of radial scars between women with and without breast carcinoma was found [15], [16].

The first study validating the association between radial scars and breast cancer with a large sample was conducted by clinical follow-up within the long-term Nurses’ Health Study in 1999 [17]. The risk of breast cancer was found to almost double with the presence of radial scar and the association still existed after adjustment for benign breast category [17]. After this, more clinical research was performed to detect cancer risk of radial scars, but the results were inconsistent. The association was attributed by some researchers to the concurrence with other benign breast lesions. However, whether radial scars confer increased breast cancer risk over other proliferative lesions remains controversial.

Radial scars are detected more frequently than ever by mammographic screening [18]–[20]. Thus, the risk assessment of radial scar and breast cancer is important to assist clinical management. We conducted this meta-analysis of clinical observational trials to investigate the relationship between radial scars and breast cancer.

## Methods

### Eligible studies

We performed a literature search limited to English language in PubMed, Web of Science and the Cochrane Library from January 2000 to December 2013 using terms “benign breast disease”, “benign breast lesions”, “radial scar”, “sclerosing lesions” and “breast cancer”. In addition, references cited in the selected articles and relevant reviews were also screened. Studies meeting the following criteria were selected: 1) evaluation of radial scars and subsequent breast cancer risk; 2) cohort or case-control study; 3) radial scars diagnosed by biopsy; 4) sufficient data for calculating odds ratio (OR) with 95% confidence interval (95%CI).

Studies were filtered from the electronic search based on title, abstract and full text by two of the authors independently. The discrepancies were resolved to finally reach a consensus. If the study populations of the articles were the same or overlapped, the one with the largest sample size was included.

### Data extraction and quality assessment

Two investigators independently extracted the following information from each eligible study: first author, publishing year, population, study design, mean follow-up time, and outcomes. Quality of the studies was evaluated using Newscastle-Ottawa Scale, [21] which was a validated technology for assessing the quality of nonrandomized studies based on three perspectives: selection, comparability and exposure or outcome.

### Statistical analysis

The strength of the association between radial scar status and breast cancer risk was measured by OR and its 95%CI. And *P*<0.05 was considered significant by Z-test. Inter-study heterogeneity was assessed by Chi^2^-based Q-test. A random-effects model (the DerSimonian and Laird method [22]) was used to pool the eligible studies first based on the hypothesis that heterogeneity existed. If the hypothesis was rejected (*P*>0.1), a fixed-effects model (the Mantel-Haenszel method [23]) was applied. Sensitive analysis and subgroup analysis were performed to find out potential origin of heterogeneity. Subgroup analyses were performed by study design, follow-up time and sample size (≥2000 and <2000). Publication bias was evaluated by the Begg test [24] and Egger test [25]. The meta-analysis was performed on Stata 12 (Stata Corporation, College Station, Texas, USA).

## Results

### Search Results

**Figure 1:**
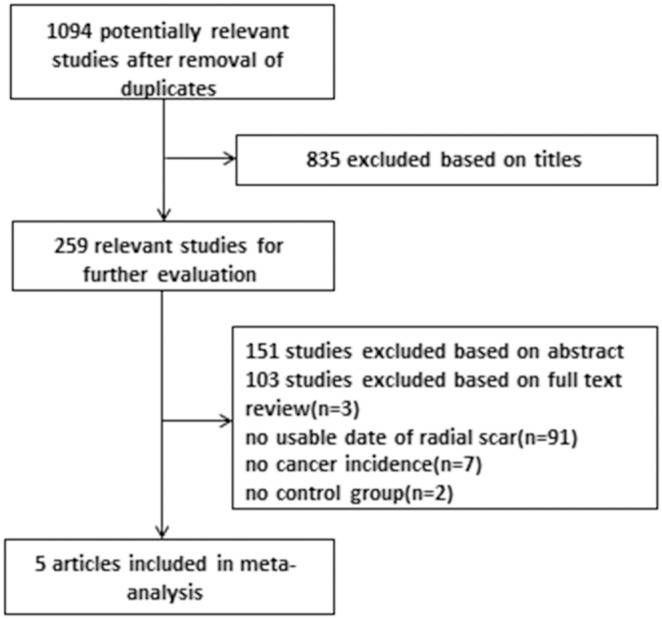
Flow chart of the selection of studies included in the meta-analysis.

The study selection is presented as a flowchart in Fig. 1. A total of 1094 records were searched in the database. Of them, 986 studies were excluded after screening the title and abstract. Finally, of the remaining 108 records, 5 records [6], [26]–[29] were included after full-text screening according to the inclusion criteria.

### Characteristics of the included studies

Baseline characteristics of the five included studies are shown in Table 1. These studies were published between 2002 and 2013. Overall, 2521 breast cancer cases and 20290 controls were involved from the eligible studies and all the five included studies originated from either America or Europe. The five studies included two nested case-control studies, two retrospective cohort studies and one case control study.

**Table 1:** Characteristics of studies about radial scars and breast cancer included in the meta-analysis.

Author	Year	Population	Design	Mean follow up time (Year)	Cases		Controls	
					Radial Scars	No radial scars	Radial scars	No radial scars
Aroner [6]	2013	NHS	Nested case-control	9.7	55	405	112	1680
Kabat [29]	2010	Ca, USA, UK	Nested case-control	15.4	13	602	21	603
Berg [28]	2008	Mayo Clinic	Retrospective cohort	15	52	669	387	8154
Sanders [27]	2006	NBC	Retrospective cohort	20.4	73	532	807	8144
Shaaban [26]	2002	British	Case-control	5.6	1	119	19	363

Notes: NHS: Nurses’ Health Studies; NBC: Nashville Breast Cohort; Ca: Canada; Cases: patients with subsequent breast cancer; Controls: patients without subsequent breast cancer.

Three of the five studies contain data of radial scar status stratified by benign breast histologic category (PDWA and AH). The Newcastle-Ottawa Scale scores for the five included studies were 8, 7, 9, 8, and 8 respectively (Table S1), indicating both the cohort and case control studies own high quality.

### Meta-analysis

**Figure 2:**
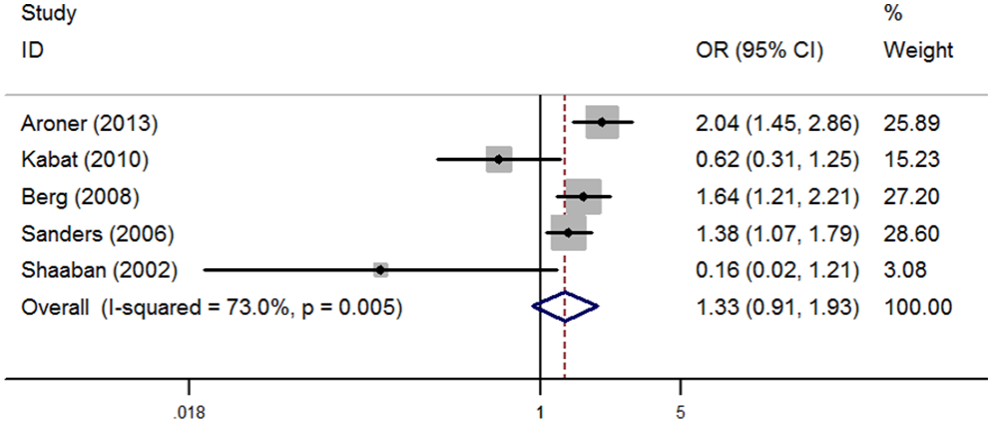
Forest plots for the association between radial scars and breast cancer risk.

A random-effects model was used to pool the results to check if heterogeneity was found in the five included studies (I^2^ = 73.0%, *P* = 0.005). The results are shown in Fig. 2. Relative to absence of radial scars, the pooled OR (95% CI) for radial scars was 1.33 (0.91, 1.93), indicating women with radial scars had increased risk of breast cancer, but which was not significant (*P* = 0.138).

In subgroup analysis (Table 2), a positive association between radial scars and breast cancer was observed among studies with a sample size more than 2000(OR = 1.6; 95%CI: 1.35, 1.89; *P*<0.001), but an adverse conclusion was found in the pooled result of studies with sample size less than 2000 (OR = 0.48; 95%CI: 0.25, 0.91; *P* = 0.025). In the subgroup analysis by study design, retrospective cohort studies show an increased risk for radial scars (OR = 1.48; 95%CI: 1.22, 1.80; *P*<0.001), but the result was not the same in case control studies (OR = 0.79; 95%CI: 0.23, 2.63; *P* = 0.696). Studies with a follow-up time >10 years also indicated radial scars could increase the risk (OR = 1.27; 95%CI: 0.87, 1.86; *P* = 0.207), but studies with ≤10 yeas follow up time hold a contrary view (OR = 0.69; 95%CI: 0.05, 8.88; *P* = 0.777).

**Table 2:** Subgroup analysis.

Subgroup	No. of studies	Odds Ratio (95%CI)	P value	I^2^	P for heterogeneity
All	5	1.33(0.91,1.93)	0.138	73.0	0.005
Case control study	3	0.79(0.23,2.63)	0.696	86.1	0.001
Retrospective cohort study	2	1.48(1.22,1.80)	<0.001	0.0	0.403
Sample size(>2000)	3	1.60(1.35,1.89)	<0.001	37.2	0.203
Sample size(≤2000)	2	0.48(0.25,0.91)	0.025	39.1	0.200
Follow up time(>10 years)	3	1.27(0.87,1.86)	0.207	68.1	0.044
Follow up time(≤10 years)	2	0.69(0.05,8.88)	0.777	84.2	0.012

**Figure 3:**
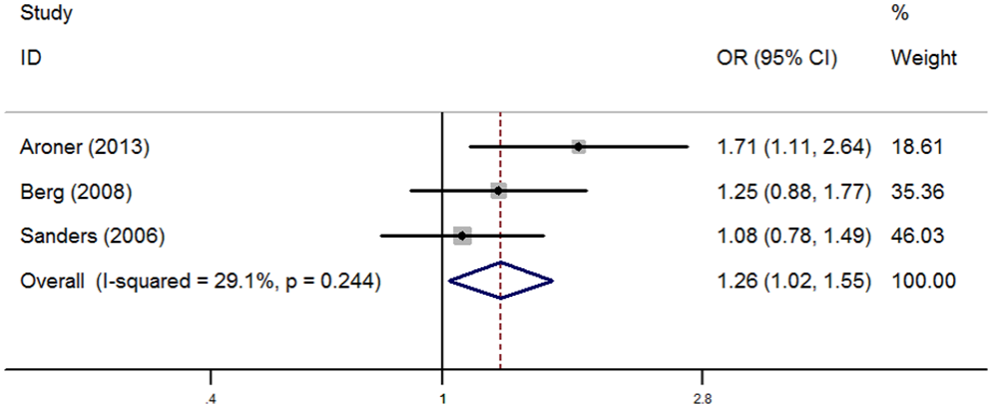
Forest plots for the association between radial scars and breast cancer risk among women with PDWA.

**Figure 4:**
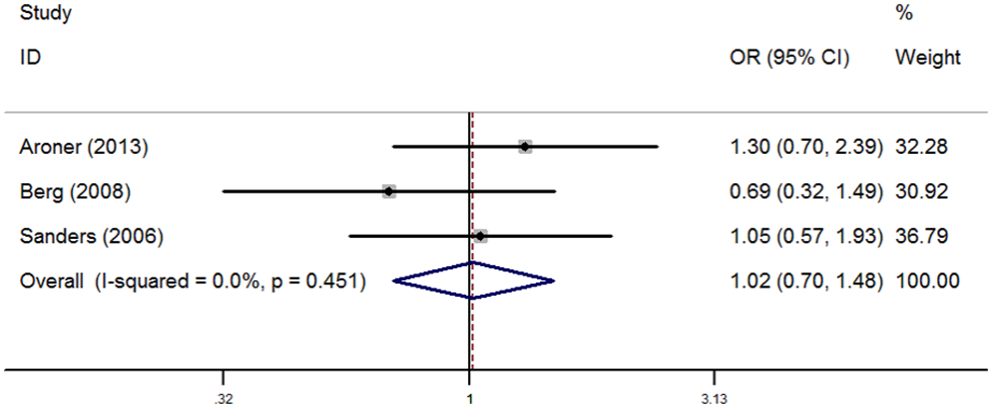
Forest plots for the association between radial scars and breast cancer risk among women with AH.

Analysis was pooled respectively for radial scars among women with PDWA and AH (Figs. 3 and 4). When analyzing radial scar status in women with PDWA, the fixed effect model was used as heterogeneity was not significant (I^2^ = 29.1%, *P* = 0.244). Relative to presence of radial scars in women with PDWA, the pooled OR for radial scars with PDWA was 1.26 (1.02, 1.55), which was statistically significant (*P* = 0.029). The pooled OR (95% CI) for radial scars in women with AH was 1.02(0.70, 1.48), which was not statistically significant (*P* = 0.923), and thus the fixed effect model was also used as there was no heterogeneity (I^2^ = 0.0%, *P* = 0.451).

Sensitivity analysis was performed by omitting studies individually from the meta-analysis, and the pooled results of the remaining studies were fairly the same (Fig. S1). We explored the source of heterogeneity in terms of sample size, follow-up time, and design of study. Analysis stratified by region was unpractical because of limitations in the data. The result showed that sample size contributed to heterogeneity.

No significant publication bias was detected in our study (*P* = 0.151 for Egger’s test; *P* = 0.806 for Begg’s test).

## Discussion

Opinions are controversial whether radial scar is related with an increased risk of breast cancer. And risks of radial scars stratified by benign breast lesions are unclear. As radial scars have been detected more frequently than before, it is urgent to solve the controversy and estimate the risk accurately. In this study, we aim to pool the results and conclude based on the published clinical observational trials.

After synthesizing the available data, radial scars were found to have 1.33 fold increased risk of breast cancer. In the results pooled from studies with sample size >2000, presence of radial scar was associated with 1.6 times breast cancer risk compared to absence of radial scar. This was contrary to the analysis of studies with sample size ≤2000. However, study with small sample size usually owns selection biases and is powerless to support or deny an association. Sample size contributed to heterogeneity in this meta-analysis, and conclusion drew from studies with large sample size was more convincing. Subgroup analysis of studies with follow-up time >10 years corroborated with the pooled analysis findings suggesting increased risk for breast cancer. In contrast however analysis of studies with follow-up time <10 years yielded a decreased risk. The incidence of breast cancer may be underestimated in studies with short follow-up time thus studies with longer follow-up time are more convincing. Retrospective cohort studies rather than case control studies which showed increased risk, this may be related to the smaller number of cases employed in the case control studies. However, case-control studies give a lower level of evidence than cohort studies because of selection bias and recall bias.

In our pooled analysis, among women with PDWA, RS increased the risk of breast cancer compared to PDWA alone. But no significant association between RS and carcinoma was noted among women with AH. Thus, the elevated breast cancer risk of radial scars could be partially attributed to the coexistence with other high-risk benign breast lesions.

There are different views regarding whether radial scar was still associated with breast cancer after stratifying benign breast. Radial scar was suggested as an independent risk factor for breast cancer [17], which was confirmed by the recent up-dated analysis of NHS. The association between RS and breast cancer among PDWA women was (RR = 1.9, 95%CI 1.1, 3.5), and among AH women was (RR = 1.7, 95%CI 0.7, 4.0), but the difference was only significant among PDWA women. On the contrary, radial scar was not suggested as an independent risk factor for breast cancer [27], [28]. Compared to PDWA and AH alone, presence of radial scar did not increase the risk significantly. Thus, the observed increased risk among women with radial scars was partially attributed to the associated proliferative disease. Difference in conclusions of these studies may be due to study sample size, criteria of participants, study design, follow-up time, and other factors.

When radial scar was present with AH together, AH was the primary concern for patients with both radial scar and AH. PDWA had a moderate association with breast cancer, and radial scars could increase the risk. Therefore, attention should be paid to patients with PDWA when radial scar was present.

Researchers also tried to find out the probable mechanism of radial scar and breast cancer. Jacobs et al found mRNA expression of some factors involved in the formation of vascular stoma was similar in radial scars and invasive breast carcinoma [30]. Iqbal et al. found a minority of radial scars had some molecular and genetic changes, which were related to breast cancer and premalignant lesions [31].

Some other studies detected radial scars and breast cancer, though not involved in our meta-analysis owing to the absence of suitable controls. A retrospective analysis of 175 patients with radial scars or complex sclerosing lesion was conducted in Northern Ireland, and over a median follow-up period of 5 years, no evidence was found to prove women with radial scars had an increased risk of subsequent breast cancer [32]. A study consisting of patients with radial scars but no atypical ductal hyperplasia (ADH) or lobular carcinoma in-situ (LCIS) upon the diagnosis was also conducted, but no evidence was found to support that RS was an independent risk factor [33].

More research in the field of radial scars and breast cancer is certainly needed, and development of radial scars stratified by benign breast lesions is essential.

There are some limitations in this study. First, the number of included studies was small. We focused on radial scars and subsequent breast cancer, but there were not enough studies focusing on this aspect. The number of studies analyzing radial scars stratified by benign breast disease was also small, and the conclusions based on them may own a bias. Second, studies exploring the presence of radial scars in breast cancer patients were not included in our study, but these studies could also supply evidence. Third, the data were not adjusted by age, age at biopsy, family history of breast cancer, menstrual status, or other risk factors. Fourth, the pooled effect of studies on radial scars among PDWA was modest, though it was significant.

In conclusion, our meta-analysis suggests that radial scars are associated with an increased breast cancer risk, and among women with PDWA, presence of radial scar could increase the risk of carcinoma. However, there are not many studies about radial scars and breast cancer risk, and thus further larger and well-designed studies are needed.

## Supporting Information

Figure S1 **Sensitivity analysis.**
(TIF)

Table S1 **Quality assessment using Newscastle-Ottawa Scale.**
(DOCX)

Checklist S1 **PRISMA Checklist of this meta-analysis.**
(DOC)
