# Supplementary figures and images for: Radial Scars and Subsequent Breast Cancer Risk: A Meta-Analysis
Source: PLoS One. 2014 Jul 14;9(7):e102503. doi: 10.1371/journal.pone.0102503 (PMC4097058; doi:10.1371/journal.pone.0102503)

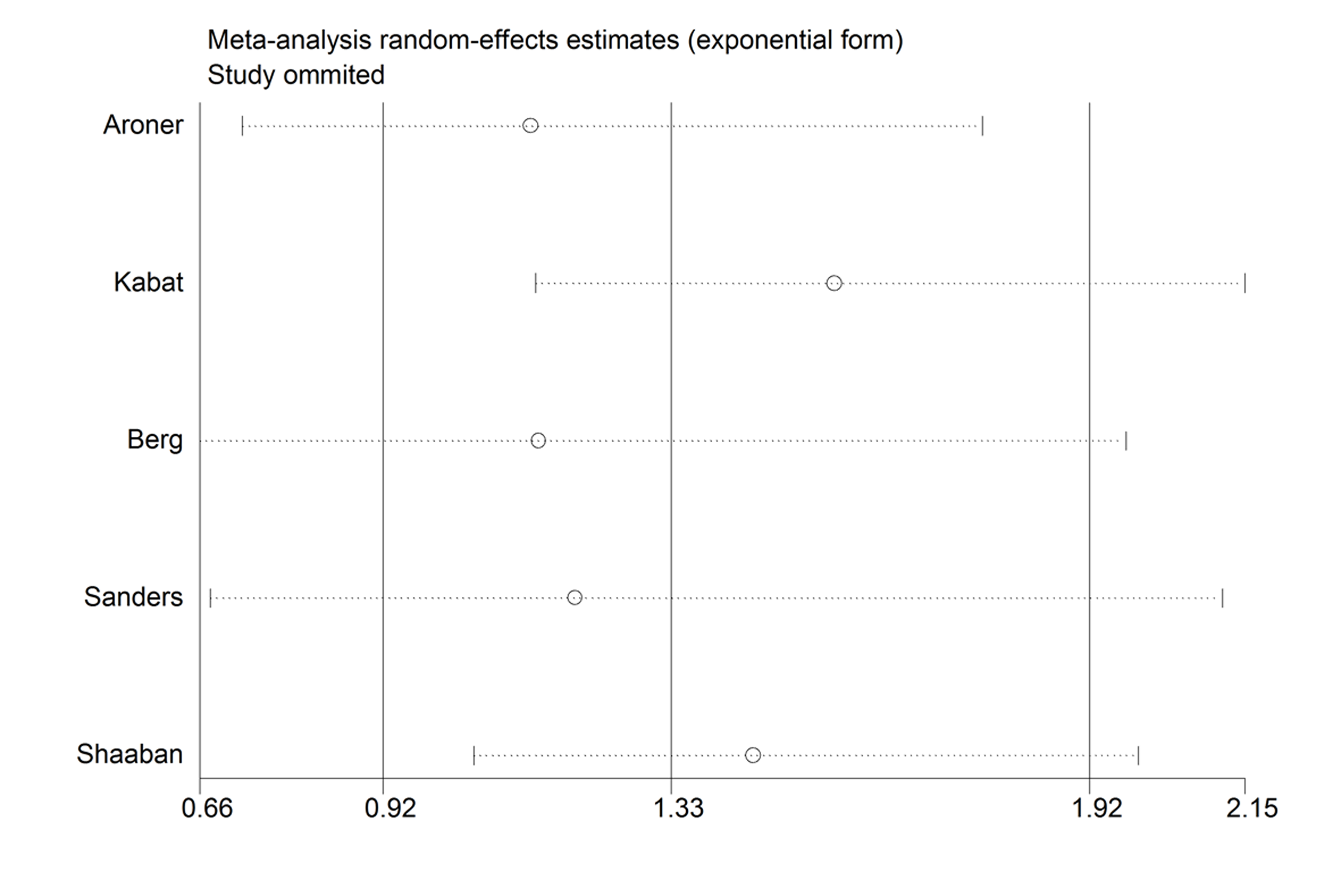

Supplement: Figure S1 — Sensitivity analysis. (TIF) [file pone.0102503.s001.tif]
