# Supplementary material for: Radial Scars and Subsequent Breast Cancer Risk: A Meta-Analysis
Source: PLoS One. 2014 Jul 14;9(7):e102503. doi: 10.1371/journal.pone.0102503 (PMC4097058; doi:10.1371/journal.pone.0102503)
Supplement: Table S1 — Quality assessment using Newscastle-Ottawa Scale. (DOCX) [file pone.0102503.s002.docx]

Table S1. Quality assessment using Newscastle-Ottawa Scale

| Study | Selection | Comparability | Exposure or outcome | Total scores |
| --- | --- | --- | --- | --- |
| Aroner(2013) | ☆☆☆☆ | ☆☆ | ☆☆ | 8 |
| Kabat(2010) | ☆☆☆ | ☆☆ | ☆☆ | 7 |
| Berg(2008) | ☆☆☆☆ | ☆☆ | ☆☆☆ | 9 |
| Sanders(2006) | ☆☆☆☆ | ☆☆ | ☆☆ | 8 |
| Shaaban(2002) | ☆☆☆☆ | ☆☆ | ☆☆ | 8 |
